# Immunosuppressive Microenvironment in Neuroblastoma

**DOI:** 10.3389/fonc.2013.00167

**Published:** 2013-06-26

**Authors:** Vito Pistoia, Fabio Morandi, Giovanna Bianchi, Annalisa Pezzolo, Ignazia Prigione, Lizzia Raffaghello

**Affiliations:** ^1^Oncology, Translational Research and Laboratory Medicine, Istituto Giannina Gaslini, Genoa, Italy

**Keywords:** neuroblastoma microenvironment, immunosuppressive mechanisms, antigen processing machinery defects, neuroblastoma derived immunosuppressive molecules, tumor microenvironment targeting

## Abstract

According to the cancer immunoediting model, the interplay between tumor cells and the host immune system is crucial for the control of tumor growth. NB is a pediatric tumor that presents with metastatic disease at diagnosis in about 50% of the cases, the majority of which have poor prognosis. In this Review article, immune escape pathways adopted by human neuroblastoma (NB) cells are reviewed. These include intrinsic defects of tumor cells such impaired expression of the HLA class I related antigen processing machinery and functional alterations of the tumor microenvironment (TM) induced by NB cell-derived immunosuppressive molecules as MICA and HLA-G. Finally, examples of therapeutic interventions targeting the TM are discussed to emphasize the concept that successful cancer treatment may be achieved using this strategy.

## Introduction

Neuroblastoma (NB) is a pediatric malignancy originating from the neural crest that presents with metastatic disease at diagnosis in approximately a half of patients (high risk cases). Children with high risk disease younger than 18 months have a 5-year survival (OS) rate of 68 ± 2%, whereas high risk patients 18 months of age or older have an OS rate of 31 ± 1% (Cohn et al., 2008), in spite of the most advanced and intensive therapeutic approaches. The bone marrow is the preferred site of NB metastases. Immunotherapy represents a new frontier that promises to improve the prognosis of high risk NB patients (Maris, 2010).

GD2 is a ganglioside expressed with high selectivity by human NB cells and a target for monoclonal antibody-based therapeutic intervention. Either murine or chimeric anti-GD2 monoclonal antibodies have been administered to NB patients in phase I–II studies with some promising results, especially in the setting of minimal residual disease (Frost et al., 1997; Yu et al., 1998; Kushner et al., 2001).

The principal mechanism whereby the ch14.18 mAbs kills tumor cells is antibody mediated cell cytotoxicity (ADCC). In ADCC, IgG1 and IgG3 antibodies bind target cells through the F(ab)2 fragment creating a bridge for effector cells, represented by macrophages, natural killer (NK) cells, and neutrophils, that bind the Fc portion of IgG1 and IgG3. Granulocyte macrophage colony-stimulating factor (GM-CSF), a potent macrophage activator, and IL-2, a strong NK cell activator, potentiate ADCC. This has led to the development of a Phase III clinical trial in which the ch14.18 was administered to NB patients in combination with GM-CSF, IL-2, and isotretinoin, a retinoid representing the gold standard treatment for NB patients in remission after myeloablative therapy and hematopoietic stem cell rescue (Yu et al., 2010). Once established that these therapeutic combinations were feasible and tolerated, the efficacy of immunotherapy (ch14.18, GM-CSF, IL-2, and isotretinoin) was compared to that of standard therapy (isotretinoin alone) in a cohort of NB patients who had a response to induction therapy and hematopoietic stem cell transplantation. Immunotherapy proved to be associated with significantly improved outcome with regard to event-free survival and overall survival (Yu et al., 2010).

In spite of these promising results, a detailed knowledge of the immunosuppressive strategies utilized by NB cells to fool the host immune system will allow to implement immunotherapeutic strategies for NB and further improve the clinical results.

The pace of tumor growth is dictated by the continuous interaction between cancer cells and the host immune system. Schreiber et al. (2011) have elaborated a conceptual model based upon sound experimental evidence that is known as “cancer immunoediting.” Cancer immunoediting operates to suppress the growth of transformed cells after intrinsic tumor suppressor mechanisms have been unsuccessful, and encompasses three sequential phases, namely elimination, equilibrium, and escape.

Both innate and adaptive immune mechanisms act in synergism during the *elimination phase* in order to counteract tumor growth before this becomes clinically apparent. The immune cell types involved are macrophages, dendritic cells (DC), NK cells, conventional CD4+ and CD8+ T cell receptor (TCR)-αβ T cells, natural killer T (NKT) cells, and TCR-γδ T cells. The mechanisms that operate in this phase include cytokines such as interferon (IFN)-γ, IFN-αβ, interleukin (IL)-12, tumor necrosis factor (TNF), cytotoxicity activating receptors such as NKG2D, and effector molecules of cytotoxicity such as TNF-related apoptosis-inducing ligand (TRAIL) and perforin (Schreiber et al., 2011).

If the elimination phase fulfils its task tumor growth is abolished. Otherwise, rare mutant cells are not destroyed and undergo the *equilibrium phase* in which they are kept in a state of dormancy by T cell dependent, but not innate, immune mechanisms (CD4+ and CD8+ TCR-αβ T cells, IFN-γ, IL-12) (Schreiber et al., 2011). It is in this phase that editing of tumor immunogenicity takes place, resulting into selection of highly immunogenic or poorly immunogenic cancer cells. The equilibrium phase may last for the lifetime of an individual leading to a definitive control of tumor growth, or allow the emergence of tumor cell variants as consequence of the immune pressure exerted continuously on genetically unstable cancer cells (Schreiber et al., 2011). Variant tumor cells (i) may be no longer recognized by the host immune system due to loss of tumor-associated antigens (TAA) or defects of the antigen processing machinery (APM), or (ii) become refractory to immune effector mechanisms, or else (iii) induce an immunosuppressive state in the tumor microenvironment (TM). These variant tumor cells undergo the *escape phase* in which they grow without the constraints imposed by the host immune system and give rise to clinically overt tumors (Schreiber et al., 2011). The mechanisms involved in this phase will be reviewed in part in the subsequent paragraphs.

From the cancer immunoediting model it is apparent that the immune system of cancer-bearing hosts may behave as double edge sword depending on the phase and the results of the editing process. In this review article we will discuss how an immunosuppressive TM is generated in NB and what are the mechanisms involved. It must be emphasized that there is more to the suppression of effective immunity in NB than a suppressive microenvironment. Among the factors that contribute are the very large tumor burdens which leads to “high zone tolerance,” as well as the fact that these patients undergo dose intensive immunosuppressive therapy.

## Tumor Microenvironment in Neuroblastoma: Infiltrating Immune Cells

The TM is a specialized *niche* that emerges during tumor progression as a result of the interaction of cancer with the host. TM is comprised of proliferating tumor cells, tumor stroma, blood vessels, and infiltrating inflammatory cells. These latter cells are molded by cancer cells to acquire a tumor-promoting phenotype (Biswas and Mantovani, 2010).

Studies on the TM in the primary tumor mass of NB patients presenting with disseminated disease at diagnosis are hampered by the treatment strategy adopted for these patients. Thus, children with high risk NB undergo a small biopsy of the primary tumor for diagnostic and prognostic evaluations. Thereafter, they are treated with a few cycles of chemotherapy and subsequently the primary tumor is surgically removed. At this time, most tumor tissue is necrotic and calcified, and therefore not amenable to studies requiring isolation of viable cell suspensions.

Histological studies have demonstrated that the stroma of high risk NB tumors is scarce as the proportion of infiltrating mononuclear cells (Shimada et al., 2001). In order to characterize the latter cells, we expanded *in vitro* cell suspensions isolated from small biopsy samples with low dose interleukin-2 (IL-2), and generated cell lines and clones (Facchetti et al., 1996). Immunophenotypic analyses revealed that the latter cells included conventional CD4+ and CD8+ T cells as well as NK cells, most of which produced pro-inflammatory cytokines such as IL-8, TNF, and IFN-γ, but also immunoregulatory cytokines such as IL-10. Many of the T and NK cell clones tested displayed cytotoxic potential (Facchetti et al., 1996).

Although these studies generated interesting information on the phenotypic and functional features of lymphocytes infiltrating high risk NB tumors, they were biased by the selection introduced by the steps of *in vitro* culture and expansion.

More recently, molecular techniques have allowed to address in depth the gene expression profiles of high risk NB tumors and provided invaluable information on the microenvironment of these malignancies. Approximately 20% of NB tumors display amplification of the *MYCN* oncogene, whereas the remaining cases are not *MYCN* amplified although displaying other structural chromosomal rearrangements (such as 3p, 4p, 11q losses and 1q, 2p, 17q gains) with or without numerical aberrations (Maris, 2010). Seeger and coworkers focused on the latter tumors and showed that these expressed at high levels genes related to macrophages, B cells, and inflammation including IL-6, IL-6R, IL-10, and TGF-β1 (Song et al., 2009). This signature was associated with dismal prognosis. Furthermore, CD68^+^ tumor-associated macrophages (TAM) coexpressing IL-6 were identified, and CD33^+^ myelomonocytic cells also expressing IL-6 were detected in metastatic bone marrow samples (Asgharzadeh et al., 2006; Song et al., 2009). *In vitro* experiments showed that NB cells stimulated peripheral blood monocytes to secrete IL-6, and TAM stimulated human NB growth in immunodeficient mice through a mechanism that depended in part on IL-6 (Song et al., 2009).

The same group has discovered that MYCN non-amplified high risk NB tumors produce the chemokine CCL2 which attracts invariant (i) NKT cells to the tumor site (Metelitsa et al., 2004). These cells represent an unconventional T cell population expressing an invariant TCRα chain (Vα24) rearranged with Jα18 and Vβ11 and recognizing self and microbial glycolipids presented by the HLA class I-like CD1d molecule (Vivier et al., 2012). Tumor cells that express CD1d are killed by iNKT cells, but this is not the case for NB cells that lack CD1d (Metelitsa et al., 2001). However, stimulated iNKT cells release IL-2 that in turn can activate NK cells, and kill monocytes pulsed with tumor lysates (Metelitsa et al., 2001). *MYCN* amplified high risk NB tumors did not produce CCL2 and consequently were not infiltrated with iNKT cells (Metelitsa et al., 2004). *MYCN* was found to down-regulate CCL2 expression, thus providing a mechanistic insight into the different composition of tumor-infiltrating lymphocytes in *MYCN* amplified vs. *MYCN* non-amplified high risk tumors (Song et al., 2007).

These findings may pave the way to the design of protocols of NB immunotherapy based upon the infusion of activated iNKT cells.

Tumor-associated macrophages are polarized toward an M2-like gene expression profile that differs from that of conventional M1-type macrophages; from a functional standpoint, M2 macrophages dampen inflammatory responses and promote tumor growth, whereas M1 macrophages activate inflammation and anti-tumor immune responses (Biswas and Mantovani, 2010). A recent study showed that localized NB tumors (that carry a favorable prognosis), stage 4s NB tumors (a special group of metastatic NB at diagnosis that affects children younger than 1 year and regress spontaneously in the majority of them), and high risk NB tumors all contained tumor-infiltrating macrophages, as assessed by immunohistochemistry. These macrophages were detected according to the expression of the M2-associated CD163 marker and were significantly more abundant in high risk than *in loco*-regional or 4s tumors (Asgharzadeh et al., 2012). Gene expression profiling experiments led to the identification of a 14 gene signature that allowed the identification of 2 discrete patient categories with a poor and a favorable outcome, respectively, based upon the score assigned. Expression of the TAM-associated genes CD33, CD16, IL-6R, IL-10, and FCGR3 accounted for 25% of the accuracy of such score (Asgharzadeh et al., 2012).

The issue of TAM sets the stage for the discussion of another major parameter of the NB microenvironment, i.e., hypoxia. Previous studies have demonstrated that hypoxia, by inducing hypoxia-inducible factor 2 (HIF-2α), maintains an undifferentiated state in human NB tumor-initiating cells (Pietras et al., 2009). Beside influencing NB biology, hypoxia affects the function of immune cells in the NB microenvironment. Thus, CCL-20 producing TAM were found to serve as an hypoxic trap for tumor-infiltrating NKT cells, and IL-15 protected antigen-activated NKT cells from hypoxia (Liu et al., 2012).

Macrophages are the major ADCC effectors in the TM. The promising results obtained with anti-GD2 therapy in combination with IL-2 and GM-CSF in NB patients with metastatic disease at diagnosis raises the question of how ADCC is affected by the NB immunosuppressive microenvironment. TAM-like macrophages were shown to phagocytose rituximab-opsonized leukemic cells more efficiently than M1 cells *in vitro*. This effect was paralleled by upregulation of the Fc receptors CD16, CD32, and CD64 in the former cells (Leidi et al., 2009). Likewise, IL-10 producing macrophages preferentially cleared early apoptotic cells (Xu et al., 2006). Thus, it is conceivable that development of TAM is associated with improved macrophage-mediated ADCC.

Limited information is so far available on two additional populations of tumor-infiltrating immune cells, i.e., T regulatory (Treg cells) and myeloid-derived suppressor cells (MDSC), in relation to NB.

Treg cells with a CD3^+^, CD4^+^, CD25^+^, Foxp3^+^ immunophenotype have not yet been investigated in NB patients; however, pre-clinical data from immunocompetent mice bearing syngeneic tumors indicate that *in vivo* depletion of Treg cells augments the efficacy of anti-NB vaccines and confers long term anti-tumor immunity mediated by CD8^+^ T cells (Croce et al., 2010; Jing et al., 2011).

**Figure 1:**
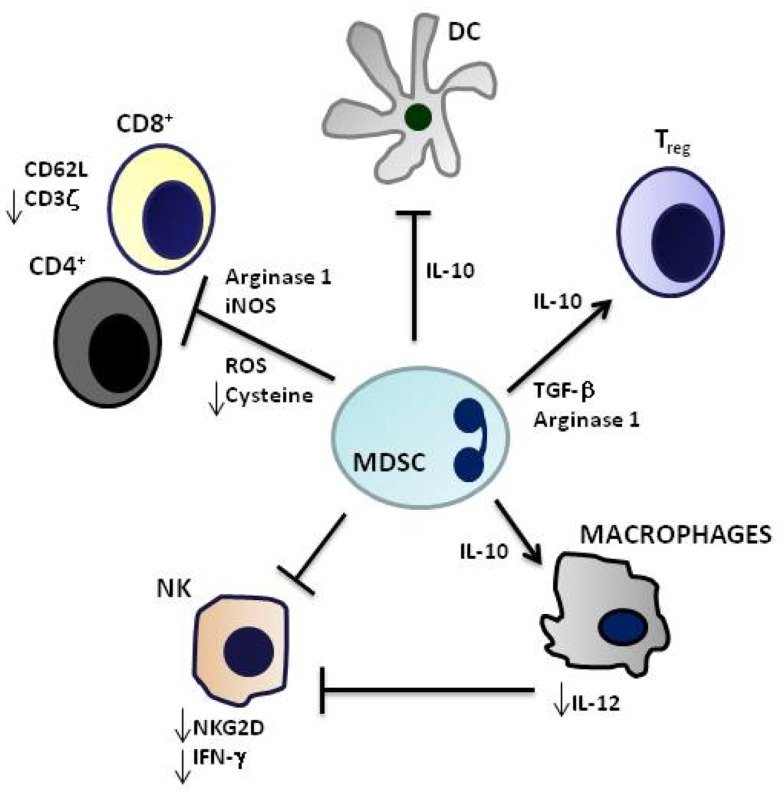
**Immunosuppressive mechanisms mediated by myeloid-derived suppressor cells**. CD4+ and CD8+ T cell activation is inhibited by Arginase 1, inducible nitric oxide synthase (iNOS), generation of reactive oxygen species (ROS) and cysteine deprivation, and induction of T regulatory cells (Treg) by IL-10 and Transforming-growth-Factor-beta (TGF-β). Innate immunity is suppressed by downregulation of dendritic cell (DC) and macrophage production of IL-12 and by inhibition of Natural Killer (NK) cell cytotoxicity. Myeloid-derived suppressor cells (MDSCs) are highly producers of IL-10 that induces Treg and Th2 cells and inhibits IL-12 production.

Immature myeloid cells are physiologically generated in the bone marrow during the process of myelopoiesis. Thereafter, these cells migrate to different peripheral organs where they differentiate into macrophages, DC, or granulocytes (Gabrilovich et al., 2012). The TM produces different soluble factors that attract immature myeloid cells to the tumor site, block their differentiation while activating and inducing them to proliferate (Gabrilovich et al., 2012). These tumor-recruited immature myeloid cells acquire immunosuppressive and tumor-promoting properties (therefore defined as MDSC), and can be distinguished in a granulocytic and a monocytic subsets differing as to immunophenotypic profiles and mechanistic activities (Gabrilovich et al., 2012) (Figure 1). Recently, MDSC have been characterized in three different NB mouse models, i.e., transgenic tyrosine hydroxylase-MYCN mice, mice xenotransplanted with human SHSY5Y cells, and A/J mice transplanted with syngeneic Neuro 2A cells (Santilli et al., 2013). MDSC, that promoted NB growth through different mechanisms, were found to be inactivated by polyphenol E, a green tea catechin, that bound the laminin receptor expressed by MDSC and promoted their differentiation to more mature granulocytic cells (Santilli et al., 2013). This study provided also a very preliminary characterization of human MDSC from NB patients expressing CD11b, CD66b, CD68, and CD33.

## Neuroblastoma Escape through Downregulation of the HLA Class I Related Antigen Processing Machinery

The APM is a molecular complex that converts endogenous protein antigens (e.g., viral antigens and TAA) into short peptides and assists presentation of the latter to CD8^+^ T cells in association with HLA class I molecules (Tanaka and Kasahara, 1998; De Verteuil et al., 2012). Polypeptides are first degraded by proteasomes in the cytosol. Constitutive 20S proteasome subunits β1, β2, and β5 are directly involved in proteolytic cleavage; in cells exposed to IFN-γ, the β1 subunit is exchanged with LMP2, the β2 subunit with LMP10, and the β5 subunit with LMP7, giving rise to the immunoproteasome, that is more efficient than the constitutive proteasome in generating immunogenic peptides (Tanaka and Kasahara, 1998; De Verteuil et al., 2012). Notably, the immunoproteasomal components are expressed constitutively in certain cell types such as DC.

Peptides derived from proteasomal digestion are structurally modified by different peptidases and subsequently bind to the transporter of antigen processing (TAP), a heterodimer composed of two different subunits named TAP-1 and TAP-2. TAP-bound peptides are translocated into the endoplasmic reticulum or the Golgi where they are trimmed further by aminopeptidases until they reach the appropriate length (8–11 mers) for binding to HLA class I proteins, formed by the association of HLA class I heavy chain (HC) with β2 microglobulin (β2m). Peptide insertion into HLA class I proteins is assisted by a loading complex composed of different chaperones, i.e., tapasin, calnexin, calreticulin, and Erp-57. Finally, the HLA class I-peptide molecular complex is exported to the cell surface (Tanaka and Kasahara, 1998; De Verteuil et al., 2012).

Antigen processing machinery abnormalities have been identified in different types of tumors and shown to cause defects in peptide generation, translocation, and loading onto β2m-HC complexes (Seliger et al., 2000). As a consequence, the latter complexes are retained in the endoplasmic reticulum and degraded by constitutive proteasomes in the cytoplasm. The few β2m-HC complexes not associated with peptides that escape degradation may reach the cell surface but their half-life is short due to instability (Seliger et al., 2000). IFN-γ treatment of APM defective cells can upregulate expression of APM components, thus restoring peptide supply to β2m-HC complexes and reversing HLA class I downregulation on the cell surface (Seliger et al., 2000).

We investigated by immunohistochemistry expression of the APM in high risk human NB tumors (Raffaghello et al., 2005). Expression of LMP7, TAP2, and β2m-free HC was never detected in these samples, but was found in pediatric adrenal medulla tested as normal counterpart of NB cells. In principle, while LMP7 defect may be compensated in part by constitutive proteasome activity, absence of TAP2 is expected to impair peptide translocation due to failure to form the TAP heterodimer, and lack of β2m-free HC hinders formation of HLA class I molecules (Raffaghello et al., 2005). Studies performed by flow cytometry with human NB cell lines confirmed the existence of APM defects, although the individual components involved were partially different (LMP2, LMP7, LMP10, TAP-1, Erp-57) (Raffaghello et al., 2005). Some of these defects were corrected by incubation of NB cell lines with IFN-γ, that caused as expected upregulation of surface HLA class I molecules. Functional studies showed that IFN-γ pre-treated NB cell lines were killed more efficiently by killer inhibitory receptor (KIR) HLA class ligand mismatched than matched activated NK cells (Raffaghello et al., 2005). These experiments indicated that IFN-γ induced upregulation of HLA class I molecules on NB cell surface, although potentially improving their detection by TAA specific HLA class I restricted CTL, inhibited elimination of tumor cells by NK cells. In this respect, it is of note that NB cells express the B7H3 costimulatory molecule which acts as a shield protecting tumor cells from NK cell-mediated lysis (Castriconi et al., 2004).

Caution is needed in extrapolating these *in vitro* studies to the clinical setting. Thus, for example, IL-2 that is now combined with anti-GD2 mAb in NB immunotherapy clinical trials in order to potentiate NK cell-mediated ADCC, is a potent inducer of IFN-γ, whose availability *in vivo* can provoke HLA class I upregulation on NB cells and therefore increase the immunogenicity of the latter cells.

## Neuroblastoma-Derived Soluble Factors as Mediators of Escape

Tumor cells release soluble molecules that inhibit immune responses thus allowing escape. Gene expression studies performed with high risk NB tumors have consistently detected transforming growth factor beta 1 (TGFβ1) and IL-10 mRNA (Asgharzadeh et al., 2006; Song et al., 2009). TGFβ1 is a potent inhibitor of T, B, and NK cell responses, as well of production of the pro-inflammatory cytokines IFN-γ, GM-CSF, and TNF. IL-10 promotes tolerogenic immune responses such as Th2 and M2 polarization. Both are produced both by tumor cells and infiltrating immune cells.

NKG2D is a cytotoxicity activating receptor expressed by NK cells, TCRγδ T cells, and some CD8^+^ conventional T cells. NKG2D binds to different ligands expressed by “stressed” cells as infected or malignant cells that include MICA, MICB, and the cytomegalovirus-related UL-16 binding proteins (ULBP) 1–5 (Raulet, 2003). We investigated expression of NKG2D ligands in primary NB cells and found that most samples expressed constitutively the transcripts of MICA and MICB, 50% of them tested positive for ULBP2 mRNA, whereas ULBP1 or 3 mRNA were never detected (Raffaghello et al., 2004). We also discovered that the MICA protein was not expressed on the surface of primary tumor cells but shed as soluble (s) molecule in serum of NB patients. MICB was identified in the cytosol but not on the surface of primary tumor cells. sMICA downregulated surface NKG2D in normal peripheral blood CD8(+) cells and decreased NK-mediated killing of MICA(+) NB cells (Raffaghello et al., 2004). These studies delineated two potential mechanisms of immune escape utilized by NB cells, (i) downregulation of surface MICA and MICB that, together with lack of expression of ULBP1 and 3 genes, renders tumor cells undetectable by NKG2D expressing immune effector cells, and (ii) shedding of sMICA, that dampens NK cell-mediated killing of NB cells. Although in our model NB cells did not express surface NKG2D ligands, this latter mechanism may contribute to tumor-associated immunodeficiency.

Galectin-1 (gal-1) is a multifunctional glycan-binding protein produced by human and mouse NB cells that suppresses T cell and DC function. In a recent study, NXS2 mouse NB cells expressing high gal-1 levels were found to be more tumorigenic in syngeneic mice than low gal-1 producing NXS2 cells. Tumors formed by the latter cells were 6–10 times more infiltrated with CD4^+^ and CD8^+^ T cells, and only supernatants from high gal-1 producing NB cells inhibited DC differentiation and induced T cell apoptosis (Soldati et al., 2012). Although these data were generated in a mouse NB model, it is conceivable that they apply also to human NB and provide another interesting example of tumor-driven immune escape. Furthermore, galectin-3 binding protein, a self-adhesive glycoprotein involved in cellular adhesion to extracellular matrix, has been shown to induce IL-6 production in bone marrow mesenchymal stem cells (Silverman et al., 2012). Since IL-6 has a well known pro-tumorigenic function, this latter cell–stromal cell interactive pathway could be a target for anticancer therapy.

HLA-G is a poorly polymorphic HLA class Ib molecule endowed with immunosuppressive activities that target all the major types of immunocompetent cells. Seven HLA-G isoforms are known (HLA-G 1–7) that generate surface and soluble molecules; in particular, HLA-G1 is a surface molecule that is cleaved to shed the soluble form, while HLA-G5 exists only as soluble molecule. The vast majority of malignant cells irrespective of their origin or histotype express HLA-G and/or release sHLA-G which contributes to immune escape (Pistoia et al., 2007).

We investigated whether human NB cells expressed HLA-G and found that such expression was low both in both primary tumors and NB cell lines. In contrast, high levels of sHLA-G were detected in sera from a subset of high risk NB patients prone to relapse (Morandi et al., 2007). We hypothesized that NB cell-derived soluble factors activated monocyte-macrophages (the cells that together with DC are the major sources of HLA-G) to release sHLA-G. Indeed, *in vitro* experiments performed by incubating circulating monocytes from normal donors with supernatants from NB cell lines demonstrated that sHLA-G production was strongly upregulated and that the immunophenotype of such monocytes was shifted toward a M2-like profile (Morandi et al., 2007). The molecular nature of these NB-derived soluble factors is unknown but we have excluded that they are related to IL-10 or TGFβ1 (Morandi et al., 2007). These results lend support to a model whereby NB cells instruct other cell types such as monocytes to produce sHLA-G.

In a recent study comparing gene expression profiles of primary vs. metastatic NB tumors we have found that the latter tumors upregulated with the highest score expression of calprotectin and HLA-G mRNA (Morandi et al., 2012). It is therefore conceivable that different mechanisms contribute to HLA-G production in human NB, i.e., instruction of other cell types by primary tumor cells and direct production of HLA-G by metastatic NB cells.

**Figure 2:**
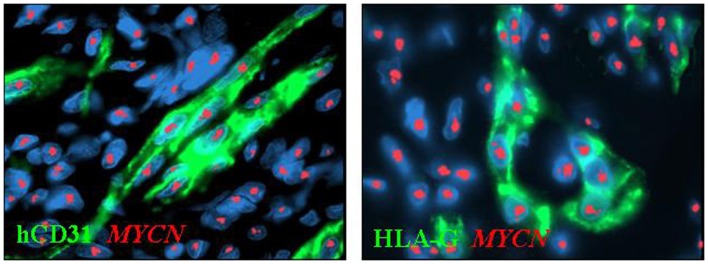
**Immunophenotypic characterization of tumor-derived endothelial cells (TEC) combined with *MYCN* FISH in orthotopic tumors formed by HTLA-230 NB cells in immunodeficient mice**. Immunofluorescence analyses show the expression of human (h)CD31 and HLA-G in TEC. Nuclei are stained with DAPI (blue). Original magnification 100×.

We have recently identified in primary and experimental NB vascular mimicry, i.e., the presence of microvessels lined by endothelial-like cells showing the same genetic abnormalities as the cancer cells from which they originate (Pezzolo et al., 2007, 2011). These tumor-derived endothelial cells (TEC) are genetically unstable and likely involved in chemoresistance and tumor progression. TEC express HLA-G (Figure 2), indicating that the transdifferentiation process whereby NB cells disguise as TEC is accompanied by upregulation of HLA-G expression (Pezzolo et al., 2011), that was virtually undetectable in primary tumor cells, consistent with previous findings (see above).

## Targeting the Tumor Microenvironment to Get Rid of Tumor

T cell-mediated immune responses are regulated by a balance between costimulatory and inhibitory signals, collectively referred to as immune checkpoints, that play a pivotal role in the maintenance of self-tolerance and the consequent prevention of autoimmunity. Expression of immune checkpoints molecules is often dysregulated by malignant transformation, leading to acquisition of immune resistance. Many costimulatory/inhibitory interactions are involved in the control of T cell responses, but two molecules in particular have attracted interest as novel therapeutic targets, i.e., cytotoxic T lymphocyte-associated antigen 4 (CTLA4 or CD152) and programed cell death protein 1 (PD-1 or CD279) (Pardoll, 2012).

CTLA4 is expressed on the surface of conventional activated CD4^+^ T helper cells where it competes with CD28 for the binding to the common ligands CD80 and CD86 expressed on the surface of antigen presenting cells. By virtue of the higher affinity for the latter ligands, CTLA4 dampens T cell activation initiated by CD28 (Rudd et al., 2009). In addition, CTLA4 is expressed constitutively on the surface of Treg cells and its engagement augments their suppressive function, whereas CTLA4 blockade causes an enhancement of T helper cell dependent immune responses (Peggs et al., 2009). Pre-clinical studies have demonstrated that CTLA4 blockade *in vivo* is followed by significantly increased anti-tumor immune response when the tumor is highly immunogenic (Leach et al., 1996). The weak immune response detected in mice bearing poorly immunogenic tumors is strengthened by combination of CTLA4 blockade with a cellular vaccine (van Elsas et al., 1999). Based upon these results, two fully humanized anti-CTLA4 monoclonal antibodies, ipilimumab and tremelimumab, have been developed and tested in clinical trials. The best results have been obtained with ipilimumab, that proved to be the first treatment showing a survival benefit for patients with metastatic melanoma (Hodi et al., 2010).

PD-1 dampens T cell activity in peripheral tissues when an inflammatory process is ongoing; PD-1 function in TM promotes immune resistance of cancer cells (Keir et al., 2008). PD-1 is expressed by activated canonical T cells and Treg cells and binds two different ligands known as PDL1 (CD274) and PDL2 (CD273) (Keir et al., 2008). Interaction of PD-1 with its ligands promotes Treg cell proliferation and can enhance their immunosuppressive activity (Keir et al., 2008). However, the major function of the PD-1/PD-1 ligand system is the control of effector T cell activation in tissues and tumors; in addition, the broad expression of PD-1 (e.g., B cells and NK cells) suggests that its blockade can upregulate the effector functions not only of T cells but also of other cell types (Keir et al., 2008). Tumor-infiltrating T cells including Treg cells express PD-1 while PD-1 ligands (especially PDL1) are upregulated on the surface of many types of malignant cells. PD-1/PD-1 ligand interactions result into suppression of anti-tumor immune responses and accelerated tumor growth (Pardoll, 2012). These pre-clinical findings have paved the way to the development of the anti-PD-1 humanized monoclonal antibody MDX 11-06, that in the first phase I clinical trial has induced some cases of tumor regression. Such regressions were observed in patients with colon, renal, and lung cancers, as well as melanoma, whose tumors contained abundant lymphocytic infiltrates, suggesting that the antibody acted by reinstating local anti-tumor immune responses (Brahmer et al., 2010).

In essence, CTLA4 and PD-1 represent two examples of successful targeting of molecules expressed in the TM in order to inhibit tumor growth. A previous study from our group demonstrated the expression of CD80 and CD86, but not PDL1, transcripts in a limited set of primary NB tumor cells, but a larger number of cases must be investigated in order to reach a definitive conclusion on this issue (Airoldi et al., 2003). Another study showed that NB cell lines expressed CTLA4 and that the latter molecule was functional (Contardi et al., 2005).

A novel mechanism associated with immunosuppressive TM is represented by post-translational modifications of chemokines operated by reactive nitrogen species. Recently it was shown that the chemokine CCL2, upon nitration, loses the ability to attract T cells but not MDSC. This leads to a *scenario* in which CTL are stopped at periphery of the tumor area whereas MDSC migrate into the core of the tumor mass where they stimulate cancer growth (Molon et al., 2011). The group that reported these findings developed a new compound named AT38 that blocks generation of reactive nitrogen species and counteracts tumor-driven immunosuppression by recruiting CTL to the tumor core (Molon et al., 2011). Clinical applications of AT38 have not yet been reported but this molecule has opened a new road to target the TM.

**Figure 3:**
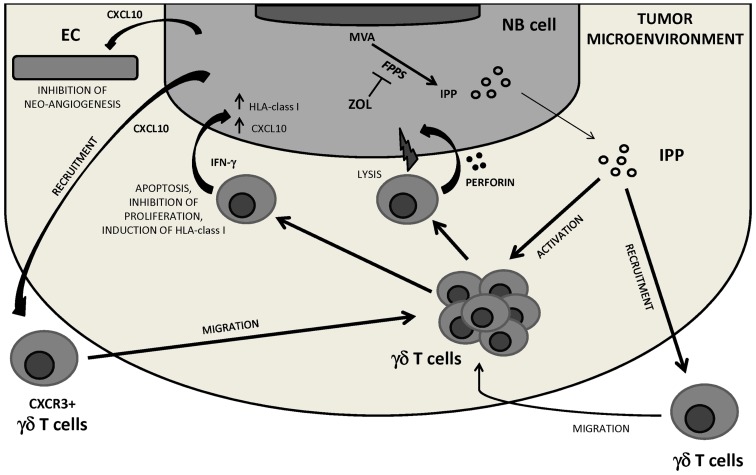
**NB tumor microenvironment perturbation by the combined treatment with ZOL and human Vγ9Vδ2 T cells**. Inhibiting FPPS, ZOL treatment induces intracellular accumulation of upstream metabolites of the MVA pathway including IPP, which attracts and activates Vγ9Vδ2 T cells. Through perforin release and IFN-γ secretion Vγ9Vδ2 T cells may be involved in NB cell killing, inhibition of NB cell proliferation, and/or induction of apoptosis. Vγ9Vδ2 T cell-secreted IFN-γ can upregulate HLA class I expression on NB cells thus increasing their immunogenicity, and induce CXCL10 expression in tumor cells. CXCL10 may exert anti-angiogenic effects by binding to CXCR3 on endothelial cells and recruit a new wave of CXCR3^+^ Vγ9Vδ2 T cells to the tumor site. FPPS, farnesyl pyrophosphate synthase; IPP, isopentenyl pyrophosphate; MVA, mevalonate; EC, endothelial cells.

A final example of therapeutic modification of the TM comes from studies performed by our group in immunodeficient mice carrying human orthotopic NB. These mice were treated with zoledronic acid (ZOL), an aminobisphosphonate that activates human TCR Vδ2 cells, or *in vitro* expanded TCR Vδ2 cells, or the combination thereof. Only the latter combination improved significantly the survival of tumor-bearing mice through a mechanism based upon (i) ZOL-driven tumor infiltration with TCR Vδ2 cells which expressed the cytotoxic granule associated Tia-1 molecule, (ii) IFN-γ production by TCR Vδ2 cells which stimulated CXCL10 expression in tumor cells, (iii) inhibition of tumor angiogenesis operate by CXCL10 that may serve as chemoattractant for continuous recruitment of TCR Vδ2 cells expressing the specific receptor CXCR3 to the tumor site (Di Carlo et al., 2013) (Figure 3).

## Conclusion

Several immune escape mechanism operated by human NB cells have been reviewed including (i) downregulation of HLA class I related APM components, that leads to defective antigen presentation and escape from NB-specific CTL recognition, (ii) expression and/or secretion of several immunosuppressive molecules, that inactivate immune effector cells, and (iii) recruitment of immunosuppressive cells, that contribute to the generation of a TM which impairs anti-tumor immune responses.

Malignant cells express antigens shared with their normal counterparts and antigens that are tumor-restricted since they arise from genetic mutations or are reactivated in cancer cells only. In order to hit selectively neoplastic cells, immunotherapy must target TAA present almost exclusively on tumor cells. A good example in this respect is represented by the above mentioned anti-GD2 monoclonal antibodies. A second pre-requisite for a candidate TAA is its expression at high level on cancer cells which reinforces interaction between BCR or TCR and the putative TAA. Finally, T cell targeted TAA must be processed into immunogenic peptides capable of associating with high affinity to MHC molecules, thus allowing strong binding of the MHC-peptide complex to TCR. Based upon these premises, highly immunogenic tumors as malignant melanoma and renal carcinoma represent good targets for immunotherapeutic approaches. NB shows a limited repertoire of genetic mutations, so that the epitope landscape may be relatively unfavorable for classic T cell-mediated immunity (Pugh et al., 2013). In conclusions, the impact of the multiple immune evasion mechanisms herein reviewed on NB biology and development of T cell-based immunotherapeutic interventions for this malignancy warrants further investigation.

## Conflict of Interest Statement

The authors declare that the research was conducted in the absence of any commercial or financial relationships that could be construed as a potential conflict of interest.

## References

[B1] Airoldi I. Lualdi S. Bruno S. Raffaghello L. Occhino M. Gambini C. (2003). Expression of costimulatory molecole in human neuroblastoma. Evidence that CD40^+^ neuroblastoma cells undergo apoptosis following interaction with CD40L. Br. J. Cancer 88, 1527–1536 10.1038/sj.bjc.6600951 12771917 PMC2377102

[B2] Asgharzadeh S. Pique-Regi R. Sposto R. Wang H. Shimada H. Matthay K. (2006). Prognostic significance of gene expression profiles of metastatic neuroblastomas lacking MYCN gene amplification. J. Natl. Cancer Inst. 98, 1193–1203 10.1093/jnci/djj330 16954472

[B3] Asgharzadeh S. Salo J. A. Ji L. Oberthuer A. Fischer M. Berthold F. (2012). Clinical significance of tumor-associated inflammatory cells in metastatic neuroblastoma. J. Clin. Oncol. 30, 3525–3532 10.1200/JCO.2011.40.9169 22927533 PMC3675667

[B4] Biswas S. K. Mantovani A. (2010). Macrophage plasticity and interaction with lymphocyte subsets: cancer as paradigm. Nat. Immunol. 11, 889–896 10.1038/ni.1937 20856220

[B5] Brahmer J. R. Drake C. G. Wollner I. Powderly J. D. Picus J. Sharfman W. H. (2010). Phase I study of single-agent anti-programmed cell death-1 (MDX-1106) in refractory solid tumors: safety, clinical activity, pharmacodynamics, and immunologic correlates. J. Clin. Oncol. 28, 3167–3175 10.1200/JCO.2009.26.7609 20516446 PMC4834717

[B6] Castriconi R. Dondero A. Augugliaro R. Cantoni C. Carnemolla B. Sementa A. (2004). Identification of 4Ig-B7-H3 as a neuroblastoma-associated molecule that exerts a protective role from an NK cell-mediated lysis. Proc. Natl. Acad. Sci. U.S.A. 101, 12640–12645 10.1073/pnas.0405025101 15314238 PMC515110

[B7] Cohn S. L. Pearson D. J. London W. B. Monclair T. Ambors P. F. Brodeur G. (2008). The International Neuroblastoma Risk Group (INRG) Classification System: an INRG task force report. J. Clin. Oncol. 27, 289–297 10.1200/JCO.2008.16.6785 19047291 PMC2650388

[B8] Contardi E. Palmisano G. L. Tazzari P. L. Martelli A. M. Falà F. Fabbi M. (2005). CTLA-4 is constitutively expressed on tumor cells and can trigger apoptosis upon ligand interaction. Int. J. Cancer 117, 538–540 10.1002/ijc.21155 15912538

[B9] Croce M. Corrias M. V. Orengo A. M. Brizzolara A. Carlini B. Borghi M. (2010). Transient depletion of CD4(+) T cells augments IL-21-based immunotherapy of disseminated neuroblastoma in syngeneic mice. Int. J. Cancer 127, 1141–1150 10.1002/ijc.25140 20039320

[B10] De Verteuil D. Granados D. P. Thibault P. Perreault C. (2012). Origin and plasticity of MHC-I associated self peptides. Autoimmun. Rev. 11, 627–635 10.1016/j.autrev.2011.11.003 22100331

[B11] Di Carlo E. Bocca P. Emionite L. Cilli M. Cipollone G. Morandi F. (2013). Mechanisms of the antitumor activity of human Vγ9Vδ2 T cells in combination with zoledronic acid in a preclinical model of neuroblastoma. Mol. Ther. 21, 1034–1043 10.1038/mt.2013.38 23481325 PMC3666635

[B12] Facchetti P. Prigione I. Ghiotto F. Tasso P. Garaventa A. Pistoia V. (1996). Functional and molecular characterization of tumor-infiltrating lymphocytes and clones thereof from a major-histocompatibility-complex-negative human tumour: neuroblastoma. Cancer Immunol. Immunother. 42, 170–178 10.1007/s002620050267 8640845 PMC11037703

[B13] Frost J. D. Hank J. A. Reaman G. H. Frierdich S. Seeger R. C. Gan J. Etalname A. (1997). A phase I/IB trial of murine monoclonal anti-GD2 antibody 14.G2a plus interleukin-2 in children with refractory neuroblastoma: a report of the Children’s Cancer Group. Cancer 80, 317–333 10.1002/(SICI)1097-0142(19970715)80:2<;317::AIDCNCR21>;3.0.CO;2-W 9217046

[B14] Gabrilovich D. I. Ostrand-Rosenberg S. Bronte V. (2012). Coordinated regulation of myeloid cells by tumors. Nat. Rev. Immunol. 12, 253–268 10.1038/nri3175 22437938 PMC3587148

[B15] Hodi F. S. O’Day S. J. McDermott D. F. Weber R. W. Sosman J. A. Haanen J. B. (2010). Improved survival with ipilimumab in patients with metastatic melanoma. N. Engl. J. Med. 363, 711–723 10.1056/NEJMoa1003466 20525992 PMC3549297

[B16] Jing W. Yan X. Hallett W. H. Gershan J. A. Johnson B. D. (2011). Depletion of CD25^+^ T cells from hematopoietic stem cello grafts increases posttransplantation vaccine-induced immunity to neuroblastoma. Blood 117, 6952–6962 10.1182/blood-2010-12-326108 21521781 PMC3128485

[B17] Keir M. E. Butte M. J. Freeman G. J. Sharpe A. H. (2008). PD-1 and its ligands in tolerance and immunity. Annu. Rev. Immunol. 26, 677–704 10.1146/annurev.immunol.26.021607.090331 18173375 PMC10637733

[B18] Kushner B. H. Kramer K. Cheung N. K. (2001). Phase II trial of the anti-G(D2) monoclonal antibody 3F8 and granulocyte-macrophage colony-stimulating factor for neuroblastoma. J. Clin. Oncol. 19, 4189–4194 11709561 10.1200/JCO.2001.19.22.4189

[B19] Leach D. R. Krummel M. F. Allison J. P. (1996). Enhancement of anti-tumor immunity by CTLA-4 blockade. Science 271, 1734–1736 10.1126/science.271.5256.1734 8596936

[B20] Leidi M. Gotti E. Bologna L. Miranda E. Rimoldi M. Sica A. (2009). M2 macrophages phagocytose rituximab-opsonized leukemic targets more efficiently than M1 cells in vitro. J. Immunol. 182, 4415–4422 10.4049/jimmunol.0713732 19299742

[B21] Liu D. Song L. Wei J. Courtney A. N. Gao X. Marinova E. (2012). IL-15 protects NKT cells from inhibition by tumor-associated macrophages and enhances antimetastatic activity. J. Clin. Invest. 122, 2221–2233 10.1172/JCI59535 22565311 PMC3366399

[B22] Maris J. M. (2010). Recent advances in neuroblastoma. N. Engl. J. Med. 362, 2202–2211 10.1056/NEJMra0804577 20558371 PMC3306838

[B23] Metelitsa L. Naidenko O. V. Kant A. Wu H. W. Loza M. J. Perussia B. (2001). Human NKT cells mediate antitumor cytotoxicity directly by recognizing target cells CD1d with bound ligand or indirectly by producing IL-2 to activate NK cells. J. Immunol. 167, 3114–3122 11544296 10.4049/jimmunol.167.6.3114

[B24] Metelitsa L. S. Wu H. W. Wang H. Yang Y. Warsi Z. Asgharzadeh S. (2004). Natural killer T cells infiltrate neuroblastomas expressing the chemokine CCL2. J. Exp. Med. 199, 1213–1221 10.1084/jem.20031462 15123743 PMC2211904

[B25] Molon B. Ugel S. Del Pozzo F. Soldani C. Zilio S. De Palma A. (2011). Chemokine nitration prevents intratumoral infiltration of antigen-specific T cells. J. Exp. Med. 208, 1949–1962 10.1084/jem.20101956 21930770 PMC3182051

[B26] Morandi F. Levreri I. Bocca P. Galleni B. Raffaghello L. Ferrone S. (2007). Human neuroblastoma cells trigger an immunosoppressive program in monocytes by stimulating HLA-G release. Cancer Res. 67, 6433–6441 10.1158/0008-5472.CAN-06-4588 17616704

[B27] Morandi F. Scaruffi P. Gallo F. Stigliani S. Moretti S. Bonassi S. (2012). Bone marrow infiltrating human neuroblastoma cells express high levels of calprotectin and HLA-G proteins. PLoS ONE 7:e29922 10.1371/journal.pone.0029922 22253825 PMC3253802

[B28] Pardoll D. M. (2012). The blockade of immune checkpoints in cancer immunotherapy. Nat. Rev. Cancer 12, 252–264 10.1038/nrc3239 22437870 PMC4856023

[B29] Peggs K. S. Quezada S. A. Chambers C. A. Korman A. J. Allison J. P. (2009). Blockade of CTLA-4on both effector and regulatory T cell compartments contributes to the anti-tumor activity of anti-CTLA-$ antibodies. J. Exp. Med. 206, 1717–1725 19581407 10.1084/jem.20082492 PMC2722174

[B30] Pezzolo A. Parodi F. Corrias M. V. Cinti R. Gambini C. Pistoia V. (2007). Tumor origin of endothelial cells in human neuroblastoma. J. Clin. Oncol. 25, 376–383 10.1200/JCO.2006.09.0696 17264333

[B31] Pezzolo A. Parodi F. Marimpietri D. Raffaghello L. Cocco C. Pistorio A. (2011). Oct-4^+^-TenascinC^+^ neuroblastoma cells serve as progenitors of tumor-derived endothelial cells. Cell Res. 21, 1470–1486 10.1038/cr.2011.38 21403679 PMC3193450

[B32] Pietras A. Hansford L. M. Johnsson A. S. Bridges E. Sjolund J. Gisselsson D. (2009). HIF-2α maintains an undifferentiated state in neural crest-like human neuroblastoma tumor-initiating cells. Proc. Natl. Acad. Sci. U.S.A. 106, 16805–16810 10.1073/pnas.0904606106 19805377 PMC2745331

[B33] Pistoia V. Morandi F. Wang X. Ferrone S. (2007). Soluble HLA-G. Are they clinically relevant? Semin. Cancer Biol. 17, 469–479 10.1016/j.semcancer.2007.07.004 17825579 PMC2200630

[B34] Pugh T. J. Morozova O. Attiyeh E. F. Asgharzadeh S. Wei J. S. Auclair D. (2013). The genetic landscape of high-risk human neuroblastoma. Nat. Genet. 45, 279–284 10.1038/ng.2529 23334666 PMC3682833

[B35] Raffaghello L. Prigione I. Airoldi I. Camoriano M. Levreri I. Gambini C. (2004). Downregulation and/or release of NKG2D ligands as immune evasion strategy of human neuroblastoma. Neoplasia 6, 558–568 10.1593/neo.04316 15548365 PMC1531660

[B36] Raffaghello L. Prigione I. Bocca P. Morandi F. Camoriano M. Gambini C. (2005). Multiple defects of the antigen-processing machinery components in human neuroblastoma: immunotherapeutic implications. Oncogene 24, 4634–4644 10.1038/sj.onc.1208594 15897905

[B37] Raulet D. H. (2003). Roles of the NKG2D immunoreceptors and its ligands. Nat. Rev. Immunol. 3, 781–790 10.1038/nri1199 14523385

[B38] Rudd C. E. Taylor A. Schneider H. (2009). CD28 and CTLA-4 coreceptor expression and signal transduction. Immunol. Rev. 229, 12–26 10.1111/j.1600-065X.2009.00770.x 19426212 PMC4186963

[B39] Santilli G. Piotrowska I. Cantilena S. Chayka O. D’Alicarnasso M. Morgenstern D. A. (2013). Polyphenol E enhances the antitumor immune response in neuroblastoma by inactivating myeloid suppressor cells. Clin. Cancer Res. 19, 1116–1125 10.1158/1078-0432.CCR-12-2528 23322899

[B40] Schreiber R. D. Old L. J. J. Smyth M. J. (2011). Cancer immnunoediting: integrating immunity’s roles in cancer suppression and promotion. Science 331, 1565–1570 10.1126/science.1203486 21436444

[B41] Seliger B. Maeurer M. J. Ferrone S. (2000). Antigen-processing machinery breakdown and tumor growth. Immunol. Today 21, 455–464 10.1016/S0167-5699(00)01692-3 10953098

[B42] Shimada H. Umehara S. Monobe Y. Hachitanda Y. Nakagawa A. Goto S. Etalname A. (2001). International neuroblastoma pathology classification for prognostic evaluation of patients with peripheral neuroblastic tumors: a report form the Children’s Cancer Group. Cancer 92, 2451–2461 10.1002/1097-0142(20011101)92:9<2451::AIDCNCR1595>;3.0.CO;2-S 11745303

[B43] Silverman A. M. Nakata R. Shimada H. Sposto R. DeClerck Y. A. (2012). A galectin-3-dependent pathway upregulates interleukin-6 in the microenvironment of human neuroblastoma. Cancer Res. 72, 2228–2238 10.1158/0008-5472.CAN-11-2165 22389450 PMC3815584

[B44] Soldati R. Berger E. Zenclussen A. C. Jorch G. Lode H. N. Salatino M. (2012). Neuroblastoma trigger an immunoevasive program involving galectin-1-dependent modulation of T cell and dendritic cell compartments. Int. J. Cancer 131, 1131–1141 10.1002/ijc.26498 22020795

[B45] Song L. Ara T. Wu H. W. Woo C.-W. Reynolds P. R. Seeger R. C. (2007). Oncogene MYCN regulates localization of NKT cells to the site of disease in neuroblastoma. J. Clin. Invest. 117, 2702–2712 10.1172/JCI30751 17710228 PMC1940236

[B46] Song L. Asgharzadeh S. Salo J. Engell K. Wu H. W. Sposto R. (2009). Valpha24-invariant NKT cells mediate antitumor activity via killing of tumor-associated macrophages. J. Clin. Invest. 119, 1524–1536 10.1172/JCI37869 19411762 PMC2689106

[B47] Tanaka K. Kasahara M. (1998). The MHC class I ligand-generating system: roles of immunoproteasomes and the interferon-gamma-inducible proteasome activator PA28. Immunol. Rev. 163, 161–176 10.1111/j.1600-065X.1998.tb01195.x 9700509

[B48] van Elsas A. Hurwitz A. A. Allison J. P. (1999). Combination immunotherapy of B16 melanoma using anti-cytotoxic T lymphocyte-associated antigen 4 (CTLA-4) and granulocyte-macrophage colony-stimulating factor (GM-CSF) producing vaccines induces rejection of subcutaneous and metastatic tumors accompanied by autoimmune depigmentation. J. Exp. Med. 190, 355–366 10430624 10.1084/jem.190.3.355 PMC2195583

[B49] Vivier E. Ugolini S. Blaise D. Chabannon C. Brossay L. (2012). Targeting natural killer cells and natural killer T cells in cancer. Nat. Rev. Immunol. 12, 239–252 10.1038/nri3174 22437937 PMC5161343

[B50] Xu W. Ross A. Schlagwein N. Woltman A. M. Daha M. R. van Kooten C. (2006). IL-10 producing macrophages preferentially clear early apoptotic cells. Blood 107, 4930–4937 10.1182/blood-2005-10-4144 16497970

[B51] Yu A. L. Gilman A. L. Ozkaynak M. F. London W. B. Kreissman S. G. Chen H. X. (2010). Anti-GD2 antibody with GM-CSF, interleukin-2, and isotretinoin for neuroblastoma. N. Engl. J. Med. 363, 1324–1334 10.1056/NEJMoa0911123 20879881 PMC3086629

[B52] Yu A. L. Uttenreuther-Fischer M. M. Huang C. S. Tsui C. C. Gillies S. D. Reisfeld R. A. (1998). Phase I trial of a human-mouse chimeric anti-disialoganglioside monoclonal antibody ch14.18 in patients with refractory neuroblastoma and osteosarcoma. J. Clin. Oncol. 16, 2169–2180 9626218 10.1200/JCO.1998.16.6.2169

